# Novel Strategy for the Recognition of Adulterant Vegetable Oils in Essential Oils Commonly Used in Food Industries by Applying ^13^C NMR Spectroscopy

**DOI:** 10.1021/acs.jafc.1c02279

**Published:** 2021-07-15

**Authors:** Eleonora Truzzi, Lucia Marchetti, Stefania Benvenuti, Annalisa Ferroni, Maria Cecilia Rossi, Davide Bertelli

**Affiliations:** † Department of Life Sciences, University of Modena and Reggio Emilia, via G. Campi 103, 41125 Modena, Italy; ‡ Doctorate School in Clinical and Experimental Medicine (CEM), University of Modena and Reggio Emilia, 41125 Modena, Italy; § Centro Interdipartimentale Grandi Strumenti, University of Modena and Reggio Emilia, Via G. Campi 213/A, 41125 Modena, Italy

**Keywords:** essential oils, counterfeit, fatty acids, DOSY, seed oils

## Abstract

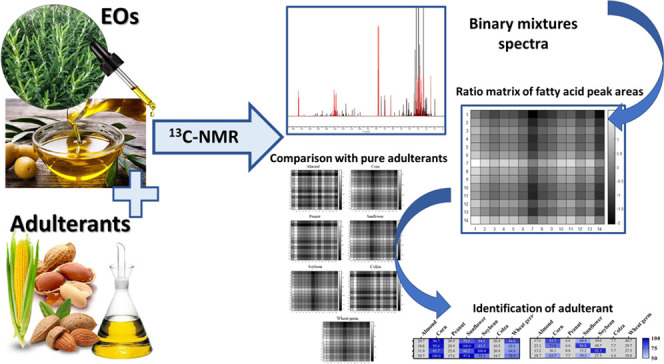

Essential oils (EOs) are valuable products commonly employed in the food industry and intensively studied as biopreservatives for the extension of food shelf-life. Unfortunately, EOs might be counterfeit to increase industrial profits. Among the possible adulterants, vegetable oils (VOs) must be considered for their characteristics and low costs. We aimed to apply nuclear magnetic resonance (NMR) spectroscopy for the detection and identification of VOs in mixtures with EOs. This innovative strategy is based on comparing the peak area ratio matrices of characteristic VO ^13^C NMR fatty acid signals with those of adulterated EOs. The identification of the VOs was achieved by calculating the matrix similarity at different confidence levels. The strategy demonstrated the capacity to efficiently recognize the presence of adulteration and the type of VO adulterant in mixtures. Thus, the method was applied to 20 commercial EOs, and VOs were detected and then identified in four samples.

## 1 Introduction

Throughout history, essential oils (EOs) have received great interest for their curative effects, and since ancient times, humans have been extracting them from aromatic plants. EOs have been employed for different purposes due to their potential in the pharmaceutical, food, cosmetic, and perfume industries. As a matter of fact, EOs exhibit several beneficial properties such as antioxidant, anti-inflammatory, antibacterial, and antiviral activities.^1−3^ The highest market demand for EOs hails from the food and beverage industries.^4^ Indeed, EOs such as peppermint, thyme, lavender, rosemary, lemon, and orange are commonly used as flavoring agents and are generally recognized as safe (GRAS). The market of EOs is expected to grow in the near future due to the increased consumer interest in “green” and natural products. Indeed, in the last decades, more and more consumers perceive organic foods as safer than conventional foods that may contain added chemical preservatives or pesticide residues.^5^ As a consequence, the consumption of organic foods has increased significantly. On the other hand, not adding food preservatives may result in a shorter food shelf-life due to microbial growth. Conventional methods of microorganism growth inhibition, such as thermal processing, cannot be applied to certain foods due to the loss of essential nutrients and sensorial properties. For these reasons, in the food industry, the application of EOs as natural inhibitors or biopreservatives with antioxidant activity against food-borne pathogens has been extensively studied.^6−9^ Moreover, EOs have exhibited pronounced effectiveness to kill pathogens and pests. The high content of lipophilic aromatic hydrocarbons provides insecticidal, ovicidal, fungicidal, and nematocidal effects to the EOs. For this reason, EOs display marked and promising features for their employment as biopesticides in agriculture.^10^

Generally, the employed EOs in most industrial fields are extracted from aromatic plants by steam distillation or cold squeezing, depending on the type of plant material. Overall, the extraction yields of EOs are rather low, resulting in high production costs. Thus, the economic potential and high market demand of EOs have led to a surge of adulteration practices of these valuable products. Besides EO devaluation due to this illicit practice, counterfeiting these products is potentially dangerous for the consumers. Indeed, EO activity and efficacy are reduced leading to an unsuitable effect in the field of application. Moreover, the fact that the actual composition is undeclared could result in allergic reactions or undesirable toxic effects in consumers. For these reasons, the quality and authentication control of EOs are currently extremely important issues.

Nowadays, common adulterations are achieved by the addition of synthetic compounds, less valuable EOs, or vegetable oils (VOs) to reach the optimal terpene composition and increase the industrial profit.^11^ The counterfeiting of EOs with VOs is one of the most common processes due to their low cost, high availability, and difficulty in identifying their presence. Indeed, the physicochemical features of EOs are not altered by the addition of VOs since the color, refractive index, and density are similar.^12^ In addition, even gas chromatography (GC), the most employed technique for EO quality control, is lacking in the ability to detect the occurrence of adulteration by VOs.^13^ Therefore, the development of alternative techniques for quality control is a challenging subject of investigation and it has been the topic of several recent studies.^14−18^ Among the recent studies on EO adulteration, the most applied technique is infrared (IR) spectroscopy coupled with multivariate analysis.^17,19,20^ However, even though the detection and quantification of an adulterant can be easily achieved, the precise identification of the VO is often difficult due to the signal overlap of similar molecules.^21^ As an example, Truzzi et al. applied IR spectroscopy in combination with chemometrics to detect and quantify VOs in EOs but was unable to discriminate the identity of the VOs employed for adulteration.^20^ Indeed, VOs are composed of triglycerides with different ratios of various fatty acids that identify the plant origin of the oil, but the spectral absorbance in IR is almost the same. Nuclear magnetic resonance (NMR) spectroscopy represents a valid and promising alternative to GC and IR spectroscopy since simultaneous quantification and identification of the type of the adulterant VO can be achieved. NMR demonstrated to be successful for quality control of food products since it is based on the recording of the nuclei electron cloud, which is different for each atom and molecule.^22−25^ Moreover, as opposed to IR spectroscopy, in NMR spectroscopy, the employment of multivariate analyses might be avoided when target signals are not overlapped and clearly assigned. Thus, a direct and punctual result can be obtained by simply integrating the peaks of interest without any manipulation of the raw data, with the exception of deconvolution when required. On the contrary, chemometrics approaches require data preprocessing and are based on probabilistic foundations.^26^

Therefore, the aim of the present study was to develop a method for the simultaneous detection and identification of VOs used as adulterants in binary mixtures with EOs by applying high-resolution ^13^C NMR. NMR alone or in combination with chemometrics was already exploited to discriminate VOs.^27−29^ However, the discrimination and identification here described are based on a novel method without the application of any multivariate analysis, which could be employed in the quality control of not only EOs but also on other food matrices where vegetable oils are present. For this purpose, binary mixtures of EOs (lavender, citronella, rosemary, and orange) and VOs (almond, corn, peanut, sunflower, soybean, colza, and wheat germ) were prepared and analyzed. The purity of the EOs was ensured prior to the analysis by ^1^H and ^13^C NMR to avoid possible interferences in the analysis.

Lavender, citronella, rosemary, and orange EOs were selected as models for their extensive use and potential application as food ingredients, biopreservatives, and biopesticides. In particular, lavender, rosemary, and orange are commonly used as flavoring agents in foods and exhibited strong antimicrobial and antioxidant activities.^30^ On the other hand, citronella is an emerging food additive and a fully recognized pest repellent. Almond, corn, peanut, sunflower, soybean, and colza seed oils and wheat germ oil were selected as adulterants for their widespread use and affordability.

After ^13^C NMR spectra acquisition of the binary mixtures, the identification of the VOs was carried out by comparing the ratio of peak areas of characteristic signals of the main fatty acids with those of pure VO used as standards. Furthermore, commercial samples of EOs were provided and analyzed to detect and identify possible adulterants.

## 2 Materials and Methods

### 2.1 Materials

Chloroform-*d* (CDCl_3_, 99.8% atom % D) and tetramethylsilane (TMS) for internal referencing were purchased from Sigma-Aldrich (Milan, Italy). Commercial samples of almond, corn, peanut, sunflower, soybean, and colza seed oils and wheat germ oil were purchased from a local marketplace. *Cymbopogon nardus* (L.) Rendle (citronella), *Lavandula angustifolia* Mill. (lavender), and *Salvia rosmarinus* L. (rosemary) EOs were purchased from Erbamea (Perugia, Italy), while *Citrus sinensis* (L.) Osbeck (orange) EO was kindly donated by L’Aromoteca (Milan, Italy). One sample of each EO was provided, and all of them were certified as 100% pure.

Furthermore, 20 commercial samples of EOs were obtained from online shops: *Mentha arvensis* L. (two samples), *Lavandula angustifolia* Mill., *Thymus vulgaris* L. (two samples), *Thymbra capitata* (L.) Cav., *Cymbopogon martinii* (Roxb.) Wats, *Mentha piperita* L. (two samples), *Eucalyptus globulus* Labill., *Origanum vulgare* L. (two samples), *Ocimum basilicum* L., *Juniperus communis* L., *Citrus limon* (L.) Osbeck (two samples), *Syzygium aromaticum* (L.) Merr. & L.M.Perry, *Salvia officinalis* L., *Chamaemelum nobile* (L.) All., *Salvia rosmarinus* L.

### 2.2 Sample Preparation

Lavender, citronella, rosemary, and orange EOs were mixed with almond, corn, peanut, sunflower, soybean, and colza seed oils and wheat germ oil to obtain binary mixtures. Starting from a stock solution at an adulterant concentration of 50% w/w, other dilutions (25, 12.5, 6.25, 3.12, 1.5, and 0.8% w/w) were obtained by adding an increasing amount of EOs. For each EO–VO combination, two dilutions were selected and a total of 56 binary mixtures were obtained for the NMR experiments. The selected dilutions for each pair of EO–VO assured that for all of the seven adulterants at least one mixture at each percent concentration was analyzed. About 50 μL of pure seed oils, pure EOs, or binary mixtures were transferred into a Wilmad NMR tube, 5 mm, Ultra-Imperial grade, L 7 in., 528-PP purchased from Sigma-Aldrich (Milan, Italy), and 550 μL of 13 mM TMS CDCl_3_ solution was added.

The commercial EOs purchased from online shops were prepared in the same manner.

### 2.3 NMR Spectroscopy and Spectrum Pretreatment

One-dimensional ^1^H and ^13^C spectra of VOs, pure EOs, binary mixtures, and unknown EOs were acquired with a Bruker FT-NMR Avance III HD 600 MHz spectrometer (Ettlingen, Germany). All of the experiments were performed at 298 K and nonspinning.

^1^H NMR experiments were acquired using Bruker sequence “zg30”; the acquisition parameters were as follows: time domain (number of data points), 131 072; dummy scans, 2; number of scans, 32; acquisition time, 4.96 s; delay time, 5 s; pulse width, 13 μs; spectral width, 22 ppm (13 204 Hz), FID resolution 0.201480 Hz; and digitization mode, baseopt. Total acquisition time was 5 min and 20 s. ^13^C NMR experiments were performed by optimizing a sequence modified to reduce the ringing effect and to completely avoid the ^1^H–^13^C coupling and NOE during relaxation. After measuring each carbon T1, delay time (D1) equal to 5 × T1_MAX_ (the longest relaxation time) was set to assure the complete relaxation of ^13^C nuclei. All of these experimental conditions were used to make the carbon integral suitable for quantitative purposes. The experiments were acquired using Bruker sequence “zgpg_pisp_f2.fas” (details are in the Supporting Information), and the acquisition parameters were consequently modified and set as follows: time domain (number of data points), 65 536; dummy scans, 0; number of scans, 256; acquisition time, 0.98 s; delay time, 10 s; spectral width, 220.87 ppm (33 333.332 Hz); FID resolution, 1.017 Hz; and digitization mode, baseopt; to use this sequence as inverse-gated, the proton decoupling power (PLW13) during recycle delay and experiment time was set to 0 db. The total acquisition time was 47 min.

To confirm the adulteration with VOs, a two-dimensional diffusion-ordered spectroscopy (DOSY) ^1^H NMR experiment was performed on the commercial EOs that resulted as adulterated. Spectral acquisitions were carried out with the standard Bruker “ledbpgp2s” pulse program using a longitudinal eddy current (LED) bipolar gradient pulse pair and two spoil gradients. Sixteen gradient steps were used for the diffusion dimension from 2 to 98% of gradient amplitude, where 65.7 G/cm was the maximum gradient intensity. The acquisition parameters were set as follows: number of scans, 32; pulse field gradient length (P30, δ), 1 ms; gradient strength (gpz6), 100%; LED delay (d21), 5 ms; and diffusion time (d20, Δ), 60 ms. Total acquisition time was 16 min.

After loading the sample into the probe, 5 min was required to achieve thermal equilibrium. Afterward, the magnetic field was locked, the probe head was tuned and matched, and, finally, the sample was shimmed. To assure the highest reproducibility, all of these procedures were automatically executed.

The baseline correction, the phasing, and the integration of NMR spectra were performed on TopSpin 3.5 software (Bruker Biospin GmbH, Rheinstetten, Germany).

The assignments for the major fatty acid peaks were carried out by a comparison of ^13^C NMR spectra with literature data.

### 2.4 VO Identification

Fourteen well-defined and characteristic signals present in all pure VOs (used as standards) and in the binary mixtures composed of EOs and adulterants were identified and integrated. Peak deconvolutions were carried out when required. These 14 resonances related to palmitic, oleic, and linoleic fatty acids were present in all of the seven VOs considered. The intensity of these signals is different in each VO since the relative abundance of each fatty acid varies considerably depending on the plant origin. In particular, the signals of the terminal and carbonylic carbons and carbons involved in double bonds in palmitic, linoleic, and oleic acid chains were selected. For each pure VO and each binary mixture, the chemical fingerprint was obtained by calculating a square ratio matrix. Specifically, the integrals of the 14 peaks in each spectrum were calculated and exported as absolute values to generate a square and symmetrical table composed of 14 rows and 14 columns. Thus, the integrals of the signals at lower chemical shifts were placed both in the first row and column and so on for all of the other signals. Then, in each cell of the table, the ratio of the corresponding peak integrals was calculated to complete the 14 × 14 matrix (196 cells). Finally, the matrices were represented as heatmaps, where the ratio of the absolute values was depicted in the grayscale range from black to white for lower and higher values, respectively.

The identification of an adulterant oil in the binary mixtures with the EOs was achieved by comparing its 14 × 14 peak ratio matrix with those of the standard pure VOs using MATLAB software (version 2020a, The MathWorks Inc., Natick, Massachusetts). In particular, for each cell, the following equation was applied
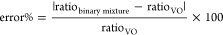


Then, the number of ratios of the total (196 – 14, which represent the number of ratios of the peaks with themself), which exhibited an error of ≤25 or 10%, depending on the considered confidence level (75 and 90%, respectively), were counted as acceptable. The percentage of likeness was calculated with the following equation
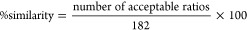


To simplify the calculations, the similarity was computed by considering the whole square matrices, even though the useful information is included in half of each matrix. The same procedure was carried out in the commercial samples that resulted in adulteration.

## 3 Results and Discussion

In the present work, the detection and identification of adulterating VOs in the counterfeiting of EOs were achieved using high-resolution NMR. Both proton and carbon NMR spectra were employed to detect the presence of VOs by focusing on the glycerol backbone signals of triglycerides. In particular, in ^1^H NMR spectra, glycerol exhibited two signals at 4.12 and 4.27 ppm, which corresponded to 2H in both positions *sn*-1′ and *sn*-3′, respectively, while the glycerol carbons were detected at 62.4 and 69.2 ppm for C1/C3 and C2, respectively.

**Figure 1:**
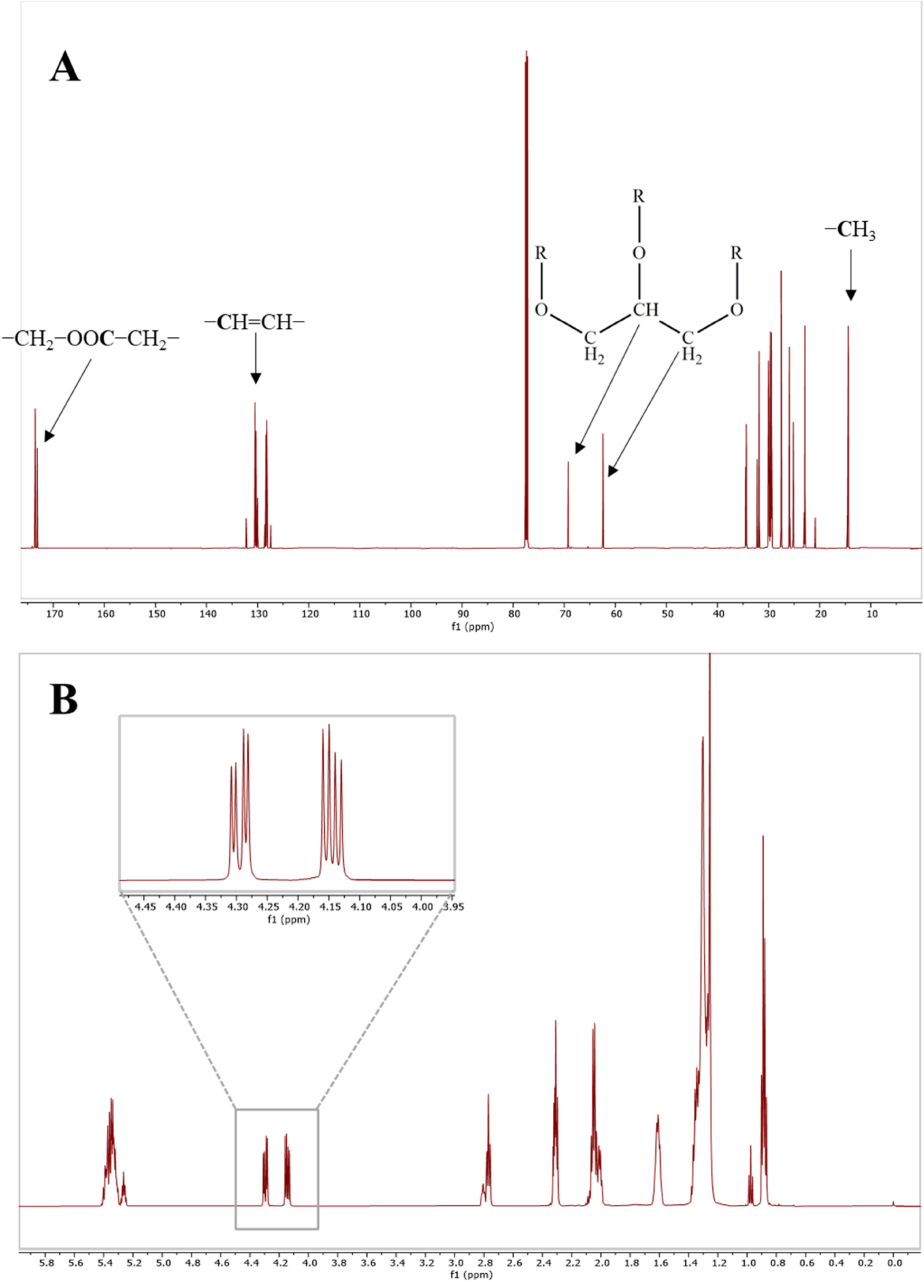
Typical spectra of soybean oil. ^13^C NMR spectra with peak assignments of the major groups of signals (A) and ^1^H NMR spectra with enlargement on *sn*-1 and *sn*-3 glycerol backbone signals (B).

Regarding the identification of the type of adulterant oil present in the EOs, ^13^C NMR was preferred over ^1^H NMR due to the ease of interpretation. Indeed, ^1^H NMR of VOs exhibited several overlapping signals with poor spectral resolution (Figure 1B). Therefore, the developed approach first involved the creation of a characteristic chemical fingerprint of each VO to identify the most representative and intense carbon signals that could be used to create a sort of chemical fingerprint library standard for the pure materials. These chemical fingerprints were then used as standard references to recognize the adulterant oils in EOs.

**Figure 2:**
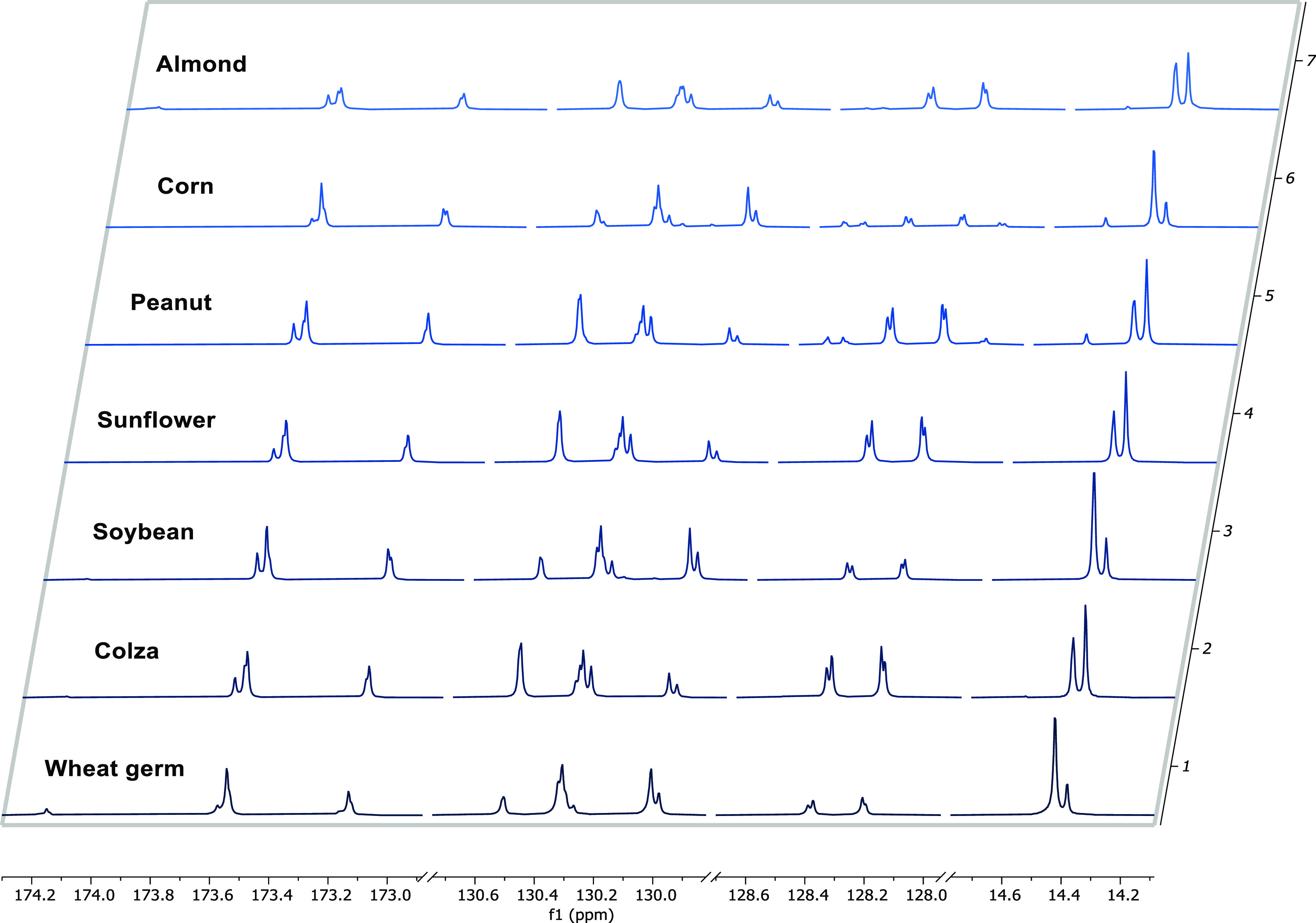
Stacked ^13^C NMR spectra of the VOs in the region of the 14 signals selected in the study.

Being that VO mixtures of triglycerides are composed of different fatty acids, especially palmitic, oleic, and linoleic acids,^31^ a typical ^13^C NMR spectrum (Figure 1A) exhibited resonances related to both glycerol and fatty acids in different regions. In particular, most of the resonances detected were assigned to the main fatty acids, palmitic, oleic, and linoleic acids. By comparing the carbon spectra of each pure VO considered in the study, a variation in the intensity of most of these signals was observed. As a matter of fact, even though the seed oils are composed of the same fatty acids, the relative abundance of palmitic, linoleic, and oleic acids varies considerably. In particular, corn, soybean, wheat germ, and sunflower oils showed high linoleic acid content, while colza, almond, and peanut oils were rich in oleic acid.^31−33^ As a consequence, this occurrence was exploited to build an efficient method for VO discrimination. Among all of the signals belonging to the three fatty acids, 14 carbon signals were selected for their optimal resolution and high variability between each VO (Figure 2). Furthermore, the carbon related to these resonances exhibited complete relaxation during ^13^C NMR spectra acquisition, guaranteeing a quantitative result. The assignment of these resonances has been performed by comparing ^13^C NMR spectra with literature data (Table 1).^34^

**Table 1:** Chemical Shifts, Relative Assignments, and Functional Groups of the 14 Selected Carbon Signals

	δ (ppm)^a^	carbon atom	functional group
1	14.390	L18	–CH_3_
2	14.433	O18	–CH_3_
3	128.204	L12, *sn*-2′	ω-6, −CH=CH–
4	128.216	L12, *sn*-1′, 3′	ω-6, −CH=CH–
5	128.383	L10, *sn*-1′, 3′	ω-9, −CH=CH–
6	128.401	L10, *sn*-2′	ω-9, −CH=CH–
7	129.994	O9, *sn*-2′	ω-9, −CH=CH–
8	130.021	O9, *sn*-1′, 3′	ω-9, −CH=CH–
9	130.285	L9, *sn*-2′	ω-9, −CH=CH–
10	130.312	L9, *sn*-1′, 3′	ω-9, −CH=CH–
11	130.523	L13, *sn*-1′, 3′	ω-6, −CH=CH–
12	173.142	L1, *sn*-2′	–CH_2_–OO**C**–CH_2_–
13	173.552	L1, *sn*-1′, 3′	–CH_2_–OO**C**–CH_2_–
14	173.595	P1, *sn*-1′, 3′	–CH_2_–OO**C**–CH_2_–

a Chemical shifts are reported with respect to TMS. L, linoleic acid; O, oleic acid; P, palmitic acid in triglycerides.

These 14 peaks can be grouped into three different regions depending on the chemical shifts that represent characterizing carbons of the fatty acids. Specifically, the most deshielded resonances represented the carbonylic carbons of fatty acids of triglycerides. These signals could be divided into two subgroups according to the resonances of the fatty acids in *sn*-1(3) and *sn*-2 positions: high-frequency and low-frequency groups. The C1 signals at lower field were assigned to palmitic and linoleic acids, where C1 of the saturated fatty acid (P1) was detected at a higher frequency in accordance with the Vlahov report.^35^

In the mid-region ranging from 128 to 130 ppm, typical resonances of the olefinic carbons were present and these signals could be differentiated based on the proximity to the ester group. As a matter of fact, the closest unsaturated carbon to the glycerol backbone (i.e., L12 and L10) shifted upfield compared to the closest unsaturated carbon to the methyl moiety (i.e., L13 and L9). Moreover, the chemical shifts of olefinic carbons were further influenced by the fatty acid position in the triglyceride. The carbon employed in a double bond (i.e., L9) closest to the carboxyl group resulted and shifted upfield in the *sn-*2 position, with respect to that in *sn-*1 (or 3). An opposite trend was noticed for the unsaturated carbons farther from the ester group.^36^

Finally, the signals at low frequencies were attributed to the terminal carbons. The resonances of terminal carbons (L18 and O18) belonging to different fatty acids could easily be distinguished since they are strongly affected by the chain length and the potential proximity to the last double bond. Indeed, the terminal CH_3_ groups of ω-6 acids have been reported to be shielded with respect to CH_3_ groups of saturated acids.^37^

**Figure 3:**
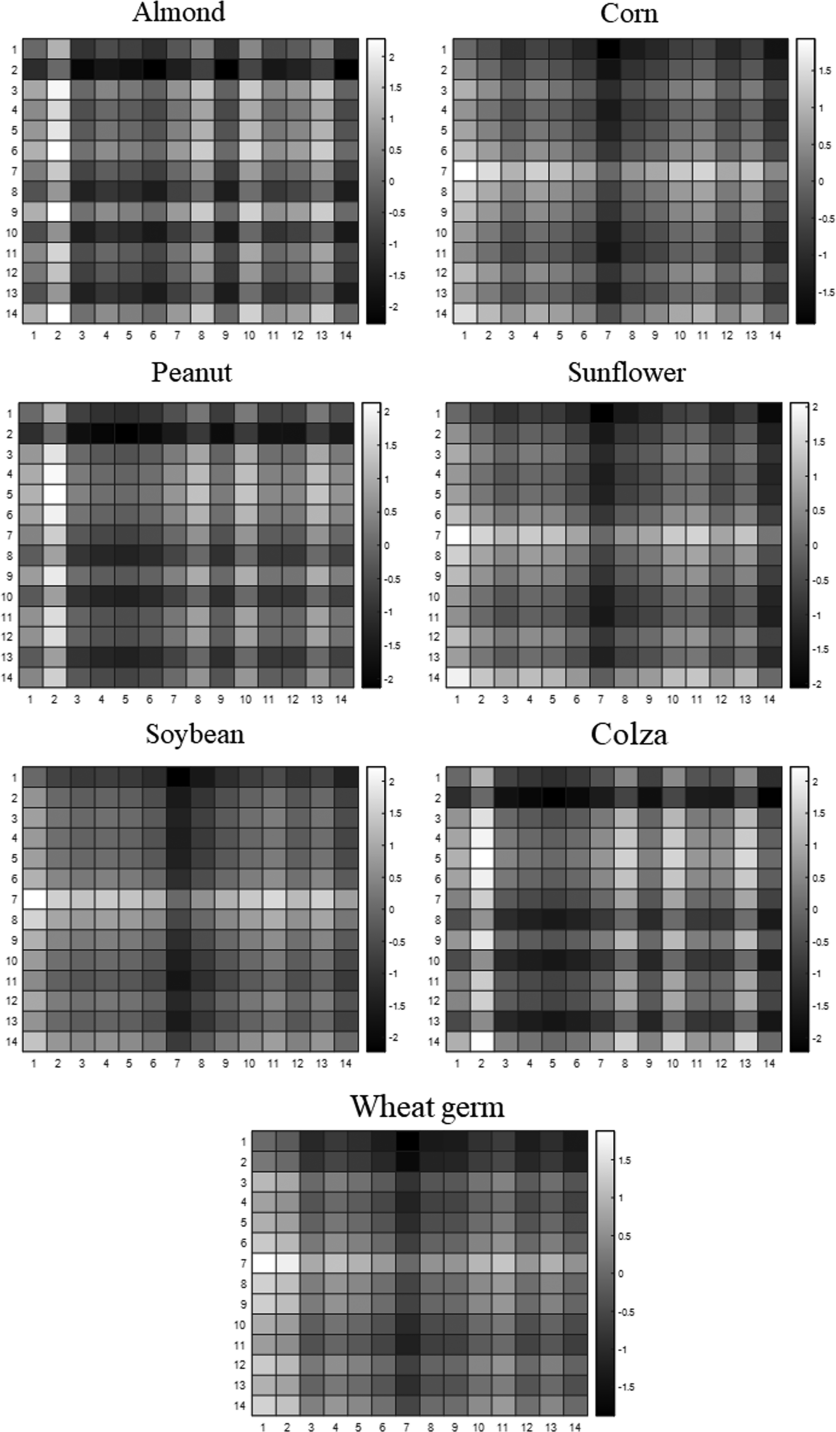
Heatmaps of standard VOs, generated by 14 × 14 matrix of peak area ratios of fatty acids.

The above-listed carbon signals, whose intensity considerably varied depending on the VO, were used to generate the chemical fingerprint for each adulterant oil. Since each VO exhibits a characteristic fatty acid composition, not only the signal intensities but also the ratios between the signals are constant. Therefore, the chemical fingerprints of pure VOs, used as standards, were obtained by integrating the 14 selected signals and calculating the ratio between each peak area. Thus, an identifier 14 × 14 matrix was attained for each VO. The ratio matrices were represented as heatmaps for convention, where the lower and higher values of the ratio were displayed in black and white, respectively (Figure 3).

Prior to the preparation of the binary mixtures, the absence of triglycerides in lavender, citronella, rosemary, and orange EOs was verified by examining their ^1^H and ^13^C NMR spectra to avoid interferences in the analysis. ^1^H NMR was preferred for purity assessment due to its higher sensitivity, related to the greater isotopic abundance of ^1^H. All of the spectra did not show any trace of the signals related to the glycerol backbone. Thus, 56 binary mixtures composed of lavender, citronella, rosemary, and orange EOs were mixed with the VOs and their spectra were acquired. In particular, for each EO–VO pair, two dilutions were analyzed, and for each VO, all of the range of percentages was assured. Concentrations from 0.8 to 50% w/w were tested as this is the most common range for adulteration. Indeed, concentrations above 50% would lead to visible detection of the VO, while concentrations lower than 0.8% would not be advantageous for profits. The glycerol backbone signals were clearly visible in the ^1^H NMR spectra even at the minimum concentration of VO in the mixtures. In the binary mixtures at 0.8% w/w, the signal-to-noise ratio was equal to 130, suggesting that the presence of adulterant oils could be detected at lower concentrations (Figure S1). Regarding ^13^C NMR spectra, the signal-to-noise ratio was decisively lower at the same concentration, equaling to 5. This value is slightly higher than the acceptable ratio for detection limit in analytical methods.^38^ Thus, as expected due to the poor isotopic abundance of ^13^C, carbon NMR could not provide reliable information at lower concentrations of adulterant oils (Figure S2).

**Figure 4:**
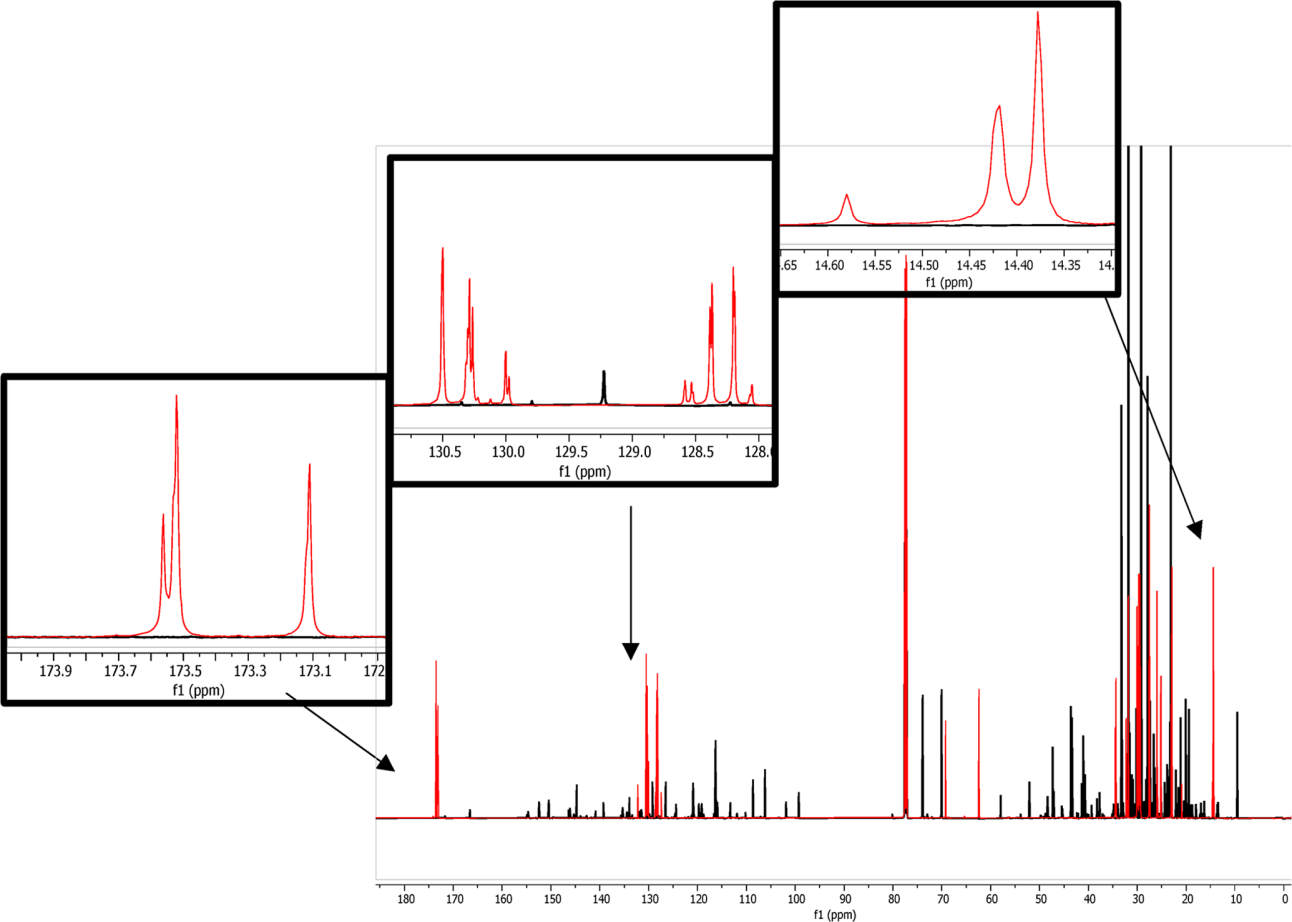
Superimposition of ^13^C NMR spectra of rosemary EO (black) and soybean oil (red).

The 14 selected carbon signals for the development of the identification strategy were well resolved and separated from the characteristic terpene peaks of the EOs in question (Figure 4).

All of the 14 typical carbon signals of the VOs were detectable in the dilution range from 6 to 50%. At a concentration of 3% w/w, some peaks disappeared. In particular, the O9 *sn*-2′ signals disappeared in soybean, wheat germ, and corn oils in mixtures, as well as L12, L10, and L9 *sn*-2′ resonances in almond oil, according to the fatty acid composition of each seed oil. The mixtures of sunflower oil showed a lack of L12 and L10 *sn*-2′ signals due to overlap with the more intense *sn*-1′(3) signals. Moreover, in peanut and colza oils, which are rich in oleic acid, the resonances belonging to L1 and L10 *sn*-1′(3) disappeared, probably due to the preferred esterification of linoleic acid in the *sn*-2′ position of triglycerides, resulting in more intense signals still detectable at a concentration of 3%. Indeed, generally, polyunsaturated fatty acids occur in the *sn*-2′ position of VO triglycerides.^39−41^ Finally, P1 *sn*-1′(3) was only missing in sunflower and almond oils where it is less abundant.^31^ At lower concentrations (1.5%), further signals disappeared.

For all 56 binary mixtures, the 14 resonances related to the three main fatty acids were integrated and the 14 × 14 ratio matrices were computed. Thus, the chemical fingerprint of the adulterant oil in each blend was generated and compared to those of standard pure VOs. The comparison and identification of the VO were attempted in all of the mixtures, and the percentage of similarity was calculated. In particular, the percentage of similarity between ratio matrices was calculated by considering the number of the peak area ratios of mixtures that matched with a peak area ratio difference lower than 25 or 10% compared to the standard VOs. Therefore, a similarity of 100% means that all 182 ratios of the matrix (14 × 14 – 14, which represent the number of ratios of the peaks with themself) exhibited a difference lower than 25 or 10% with the standard VO.

In the case of binary mixtures at a concentration of 3%, the matrices were reduced and the peak area ratios of the missing resonances were eliminated. However, due to the lack of most signals, the recognition was not achieved on the samples below 1.5% of adulterants, and for this reason, the results were not reported.

**Figure 5:**
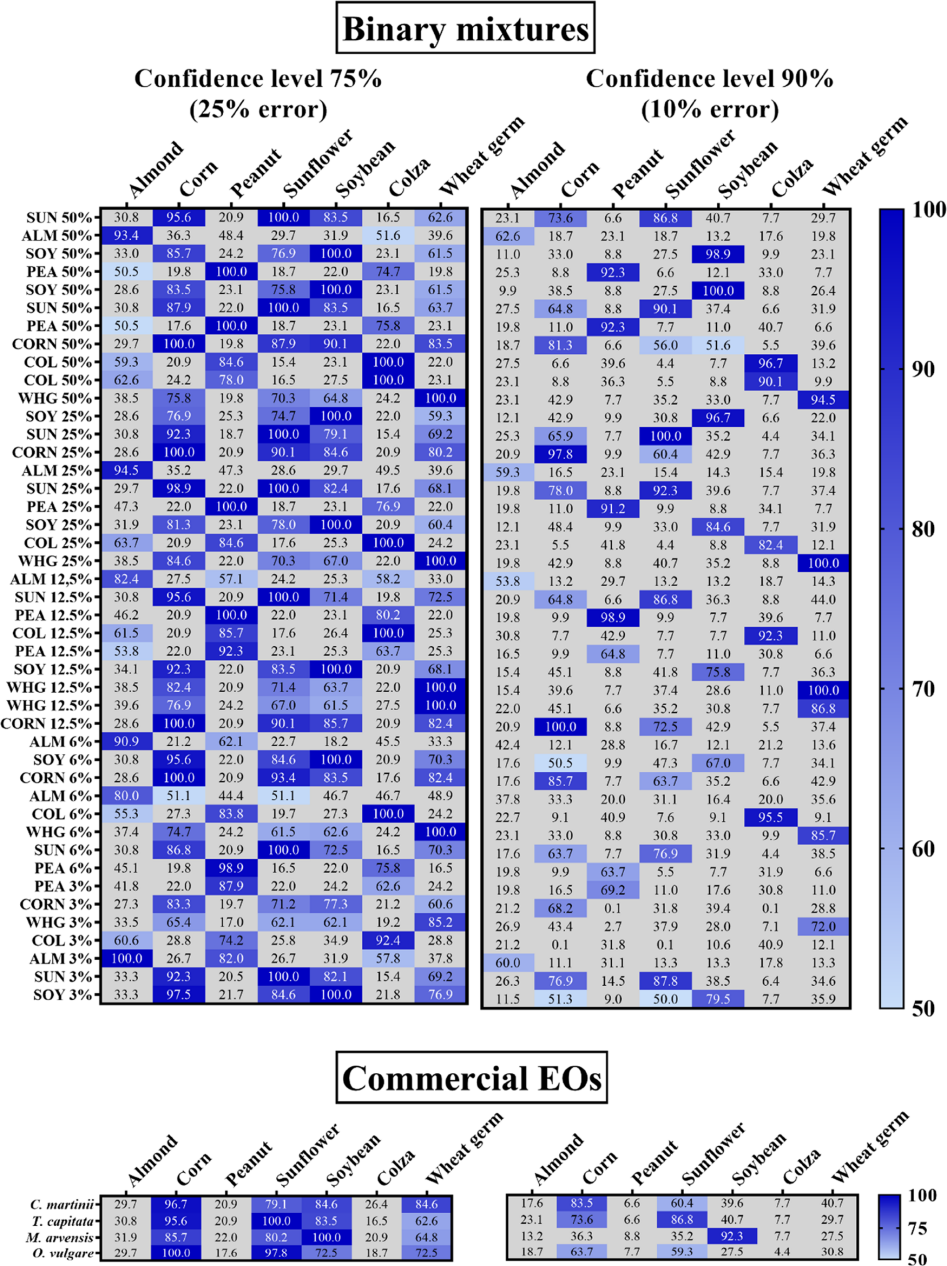
Heatmaps representing the similarity results for the analyzed binary mixtures and adulterated commercial samples at the confidence levels of 75 and 90%. ALM, almond; COL, colza; PEA, peanut; SOY, soybean; SUN, sunflower; WHG, wheat germ.

As shown in Figure 5, the identification method successfully recognized the adulterant oil in all of the tested binary mixtures from 3 to 50% w/w of VO concentration. The percentages of similarity were higher by accepting an error of matching of 25%, especially at low concentrations of adulterants (i.e., 6 and 3%), as expected. As a matter of fact, the method failed in some cases in the identification accepting an error lower than 10%. On the contrary, at this confidence level, increased accuracy in VO recognition was observed, achieving a greater deviation of the percentages of similarity in VOs with similar fatty acid compositions. Indeed, by considering a confidence level of 75%, all of the EOs adulterated with corn, sunflower, or soybean showed a high likeness with each other, making difficult it to distinguish between the VOs. As can be noticed in Figure 5, by increasing the confidence level to 90%, the mixtures composed of corn oil were not recognized as adulterated with soybean and the similarity to sunflower oil was less pronounced, and *vice versa*. By further increasing the confidence level to 95% and reducing the acceptable error to 5% (data not shown), the correct identification of the VO gave rise to a decreased percentage of likeness with the exception of the binary mixtures with 50 and 25% adulterant concentrations. In this case, the weakening of recognition is ascribed to a poor signal-to-noise ratio and imprecise signal deconvolutions at low adulterant percentages. As a consequence, by decreasing the margin of error to 5%, several peak area ratios did not match with the standard VO, leading to lower similarity percentages.

These results demonstrated the capability of the developed method to recognize the adulterant oil in EO mixtures. As a matter of fact, the correct identification was achieved for all of the prepared mixtures in the concentration range of 3–50% w/w of the adulterant in the tested confidence levels, without any difference with respect to the EO in analysis. A confidence level of 75% was demonstrated to be less selective for the determination of the adulterant but more sensitive and accurate at low adulterant concentrations. On the contrary, the 90% confidence level was more precise in identifying which VO was present but less sensitive to the presence of the adulterant in general. The identification of VOs has already been tested exploiting the potential of NMR analysis. Popescu et al. achieved a good differentiation of VOs, demonstrating that linoleic, oleic, linolenic, and free fatty acids were the most important discriminant variables.^28^ On the contrary, Zamora et al. argued that a better differentiation of VOs is obtained by considering minor compositional components, which emerged by fractionating the whole oil by column chromatography.^42^ However, all of these outcomes were attained by applying principal component analysis on ^1^H and ^13^C NMR resonance intensities of VOs.

The advantages of our approach relied on obtaining successful results without requiring any data preprocessing or multivariate analyses, which are based on probabilistic foundations. Moreover, our promising outcomes were achieved by merely considering the signals of three common fatty acids in pure VOs without any pretreatment. Therefore, the feasibility and the strength of the novel developed method can be affirmed.

**Figure 6:**
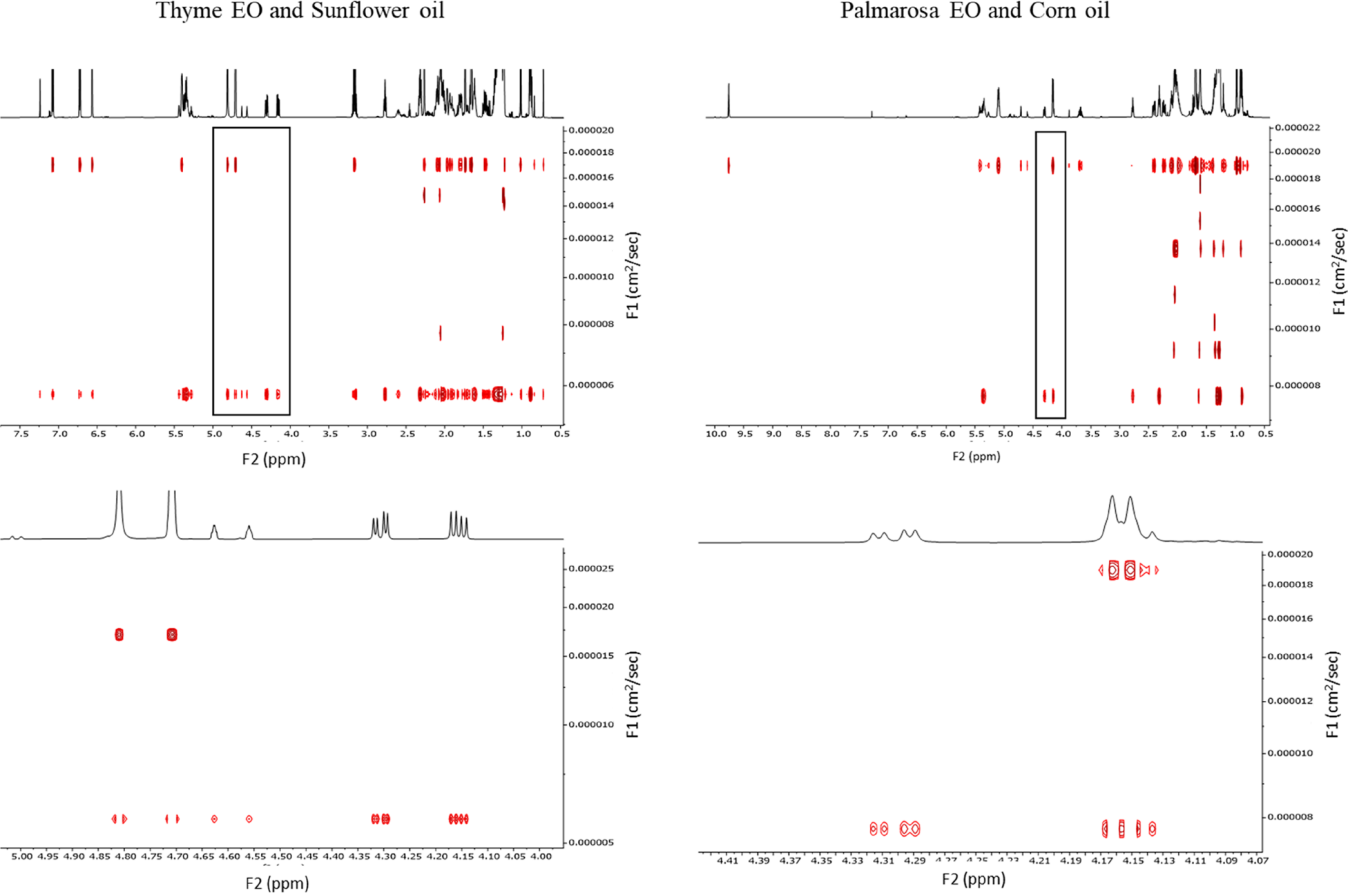
Entire (top) and cross-sectional (bottom) DOSY ^1^H NMR spectra of commercial samples of thyme and palmarosa EOs that resulted in counterfeit.

The validated method was then applied to commercial samples of EOs purchased on the internet to detect possible adulterations. The analysis of these samples was carried out in triplicate to assure the results. Among the 20 EOs, derived from different plant species and brands, the samples of *Thymbra capitata* (thyme), *Cymbopogon martinii* (palmarosa), *Mentha arvensis* (mint), and *Origanum vulgare* (oregano) resulted in counterfeit since typical signals of the glycerol backbone were identified in both ^1^H and ^13^C NMR spectra. The recognition method revealed that thyme was adulterated with sunflower, mint with soybean, and palmarosa and oregano with corn with a certainty of recognition that ranged from 96.7 to 100% and 63.7 to 92.3% at the confidence levels of 75 and 90%, respectively (Figure 5). To assure the presence of the triglycerides of seed oils rather than free fatty acids or other esters in the commercial samples of EOs, DOSY ^1^H NMR spectra were acquired. DOSY experiment is a well-known method for counterfeit identification based on the different diffusion levels of compounds in a solution.^43,44^ The pulsed field gradient used in the acquisition can be used to measure the translational diffusion of the compounds, which is influenced by the molecular weight. Indeed, high-molecular-weight molecules diffuse slower than low-molecular-weight molecules, resulting in a different position along the *y* axis of the spectrum (F1). As an example, in Figure 6, the DOSY spectra of thyme and palmarosa are displayed.

The signals of the glycerol backbone (2H at 4.282 ppm and 2H at 4.130 ppm) were used to identify triglycerides in the oils. For both the adulterated EOs, DOSY signals can be divided into two main groups based on the diffusion rate coefficients (*D*) of the compounds in the sample. Specifically, triglycerides diffused more slowly, while terpenes in the EO, being the lightest compounds in the mixture, displayed a higher *D* and diffused more rapidly. In the case of adulterated palmarosa, three more groups of compounds could be identified. Indeed, within terpenes and triglycerides, molecules with medium molecular weight exhibited *D* equal to 2.5 × 10^–5^, 3.1 × 10^–5^, and 3.5 × 10^–5^ cm^2^/s. Being that corn is the richest source of tocopherols among the VOs, these signals might be related to them in their free form or esterified with fatty acids.^45^ The identity of these signals was also confirmed by performing a DOSY experiment on our corn oil sample, demonstrating that these signals belong to the VO. Even though sunflower has been reported as a phytosterol source, no signals were detected, probably due to a lower concentration of the adulterant in the commercial thyme with respect to palmarosa.

In conclusion, this newly developed strategy demonstrated feasibility and efficiency to identify and recognize VOs used as adulterants in EOs. In particular, the detection of the presence of VOs could be achieved from the minimum concentration of 0.8% w/w of the adulterant both in proton and carbon spectra. The VO’s identification method was shown to be effective in all of the cases by accepting a margin of error of 25%, without employing multivariate analyses. The simplicity of the proposed approach could be exploited in fraud detection of different food matrices containing VOs. To the best of our knowledge, this method could represent a valid alternative to other conventional techniques, such as chromatographic methods or IR spectroscopy. Further studies will be carried out to improve the application of NMR spectroscopy in the quantification of VOs in these valuable products in the range of 0.8–50% w/w of adulterant content, being one of the most exploited adulteration practices.

## Supporting Information Available

^1^H NMR spectrum enlargement on glycerol backbone signals of a binary mixture and ^13^C NMR spectrum enlargement on glycerol backbone signals of a binary mixture (PDF)

## Abbreviations

DOSY: diffusion-ordered spectroscopy
EO: essential oil
GC: gas chromatography
IR: infrared
LED: longitudinal eddy current
NMR: nuclear magnetic resonance
TMS: tetramethylsilane
VO: vegetable oil
