# Supplementary material for: Novel Strategy for the Recognition of Adulterant Vegetable Oils in Essential Oils Commonly Used in Food Industries by Applying 13C NMR Spectroscopy
Source: J Agric Food Chem. 2021 Jul 15;69(29):8276–86. doi: 10.1021/acs.jafc.1c02279 (PMC8389833; doi:10.1021/acs.jafc.1c02279)
Supplement: Supplementary file 1 — jf1c02279_si_002.pdf [file jf1c02279_si_002.pdf]

ZGPG_PISP_F2.fas SEQUENCE

```
 ;zg_pi
;avance-version(07/03/21)
;1D sequence
;a weak 2-5% gradient may also be added after the pi pulse
;$OWNER=nmr
#include<Avance.incl>
#include <Delay.incl>
"d14 = 1.0m"
"acqt0=-p1*2/3.1416"
"d11=30m"
"DELTA=d1-100m"
"p20=1500"
1 ze
d11 pl12:f2
2 30m do:f2
10u pl13:f2
100m cpd2:f2
DELTA
"l9=2*((l1)%2)"
1u iu1
if "l9==0" goto lab3
(p20:sp12 ph1):f1
d14
4u do:f2
lab3, 4u
d11 pl1:f1 pl12:f2
p1 ph2
go=2 ph31 cpd2:f2
30m do:f2 mc #0 to 2 F0(zd)
exit
ph1=0
ph2=0 0 1 1 2 2 3 3
ph31=2 0 3 1 0 2 1 3
;pl1 : f1 channel-power level for pulse (default)
;p1 : f1 channel-high power pulse (90deg)
;pl12: f2 channel - power level for CPD/BB decoupling
;sp1: f1 channel - shaped pulse (180degree inversion)
;spnam2: Crp60,0.5,20.1
;d1 : relaxation delay; 1-5*T1
;d11: delay for disk I/O (30ms)
;d14 : pi delay
;NS : 1*n, total number of scans: NS*TD0
;cpd2: decoupling according to sequence defined by cpdprg2
;pcpd2: f2 channel - 90 degree pulse for decoupling sequence
;plw13=0 for quantitative purpose without NOE
```

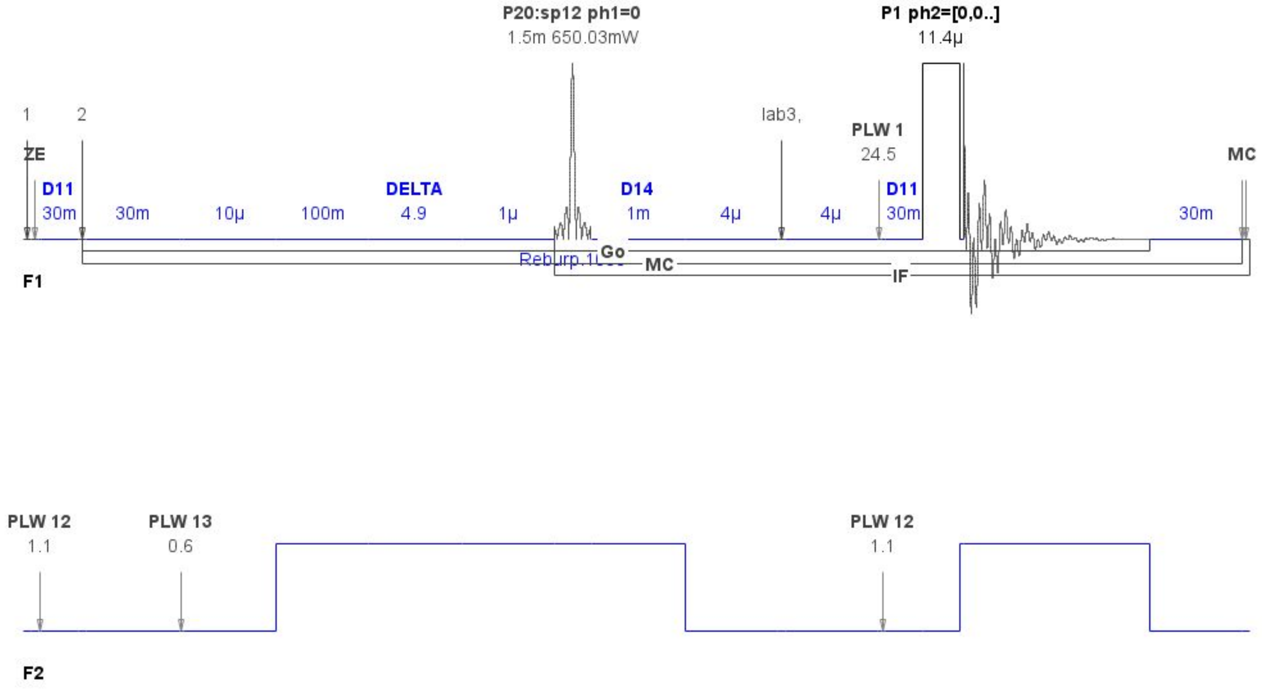

P20:sp12 ph1=0
1.5m 650.03mW
P1 ph2=[0,0..]
11.4µ
1
2
lab3,
PLW 1
24.5
ZE
MC
D11
30m
30m
10µ
100m
DELTA
4.9
1µ
D14
1m
4µ
4µ
D11
30m
30m
Go
MC
IF
F1
PLW 12
1.1
PLW 13
0.6
PLW 12
1.1
F2

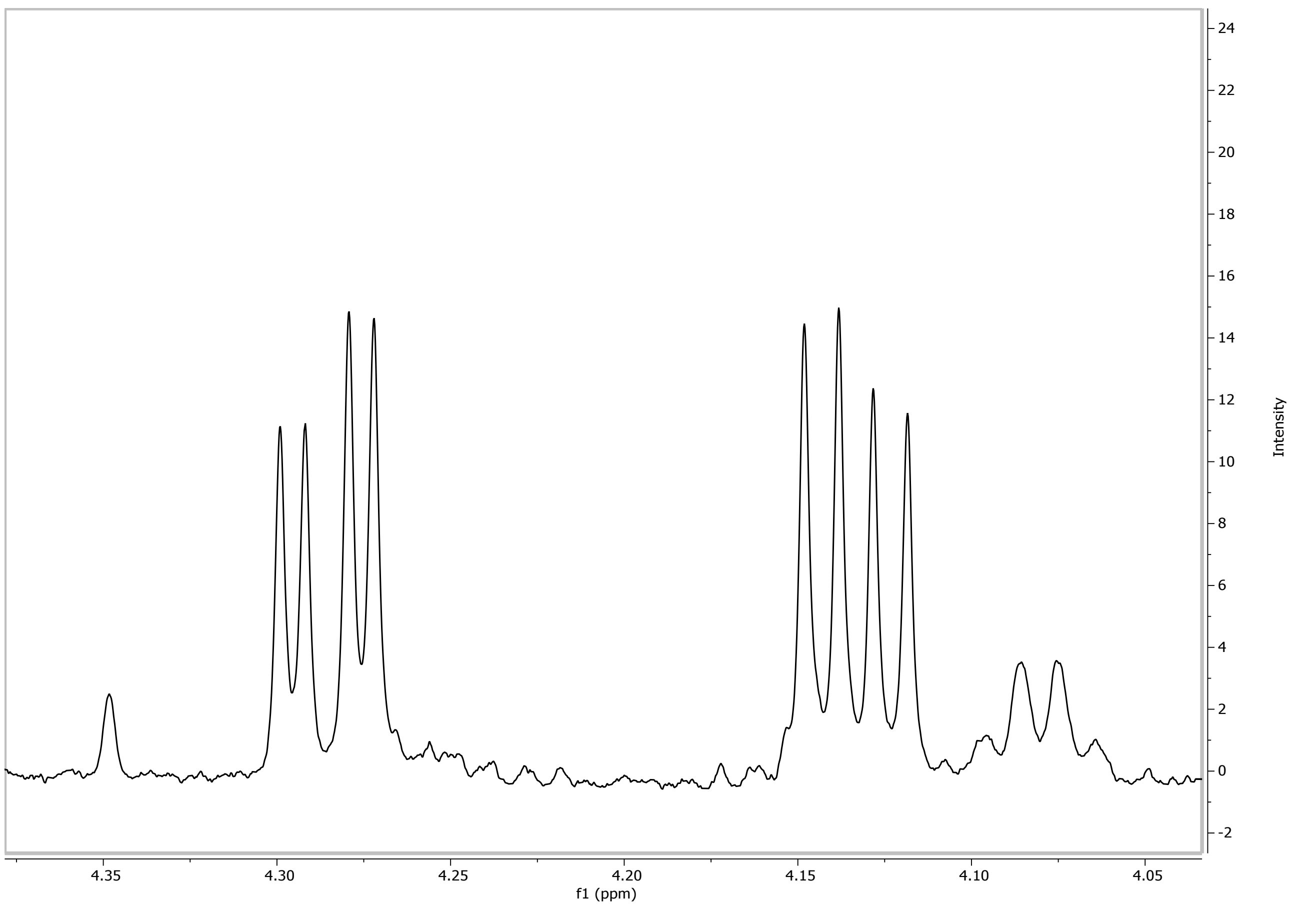


**Figure S1.** $^{1}$H-NMR spectrum enlargment on glycerol backbone signals of a binary mixture composed by rosemary EO and peanut at the concentration of 0.8% w/w.

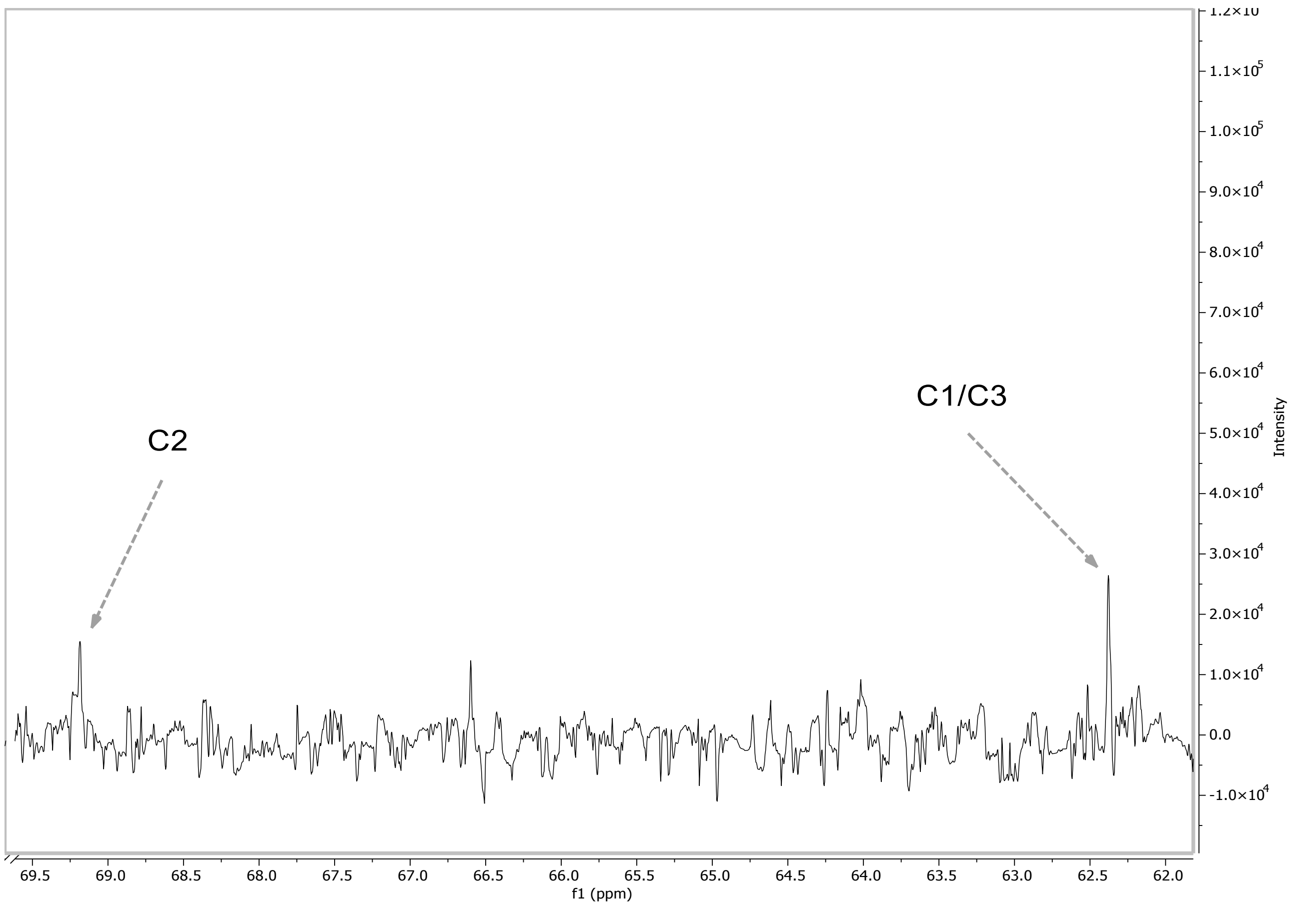


**Figure S2.** $^{13}$C-NMR spectrum enlargment on glycerol backbone signals of a binary mixture composed by rosemary EO and peanut at the concentration of 0.8% w/w.
